# Impact of dopants on X‑ray attenuation of 3D‑printed polymers for mammography and tomosynthesis spectra

**DOI:** 10.1002/acm2.70739

**Published:** 2026-08-06

**Authors:** Adrián Belarra, Irene Hernández‐Girón, María Castillo‐García, Peter Homolka, Margarita Chevalier

**Affiliations:** ^1^ Medical Physics, Radiology and Rehabilitation Department, Facultad de Medicina Universidad Complutense de Madrid Madrid Spain; ^2^ School of Physics University College Dublin (UCD) Dublin Ireland; ^3^ Center for Medical Physics and Biomedical Engineering Medical University of Vienna Vienna Austria

**Keywords:** 3D‐printing, digital breast tomosynthesis, digital mammography, effective attenuation coefficients

## Abstract

**Background:**

3D‐printing enables the production of anthropomorphic breast phantoms for assessing image quality in digital mammography (DM) and breast tomosynthesis (DBT). 3D‐printing materials must be selected carefully to mimic breast tissue attenuation for clinical spectra.

**Purpose:**

This study investigates the impact on the effective X‐ray attenuation coefficient (*µ_eff_*) of polymer formulation and dopants (e.g., colorants) used in 3D‐printing materials. It also analyses the impact on their equivalent glandular ratios (*BR*) relative to commercial materials (CIRS).

**Methods:**

Five base polymers (PLA, ABS, PETG, HIPS, RESIN) were selected among the catalogue of five manufacturers. A collection of nineteen variants (base polymer + color) was 3D‐printed as step‐wedges with thicknesses (*t*) from 0.5 to 5.5 cm and imaged on a clinical mammography system (DM: W/Rh with the anti‐scatter grid, DBT: W/Al without grid, at 27, 29 and 31 kVp). The *µ_eff_(t)* values were derived from the mean pixel value measured at each step of the logarithmically transformed images. As reference, *µ_eff_* values were measured for the CIRS materials with well‐known *BR*. A two‐parameter empirical model was used to fit the values as: *µ_eff_(t) = µ_0_ /(1+kt)*, where *µ_0_* corresponds to *µ_eff_* at thickness tending to zero, and *k* is the decrease rate with thickness due to beam hardening and scatter. The impact of dopants on polymer attenuation was evaluated by analyzing both *µ_eff_* and *µ_0_* values. Based on *µ_0_–BR* relationships of CIRS materials, the corresponding *BR* values were computed for the investigated materials.

**Results:**

For all acquisition conditions, some color variants of 3D‐printed materials with the same base polymer exhibit noticeable differences in *µ_eff_*, independently of the thickness, particularly for DM. For this modality, the maximum differences within *µ_eff_* values of materials sharing polymer base decreased by 62% for PLA‐based, 44% for PETG‐based and 30% for ABS‐based materials, when the thickness increases from 1.5 to 5.5 cm. For DBT, a lower variability of *µ_eff_* values was observed. The materials best mimicking purely glandular and adipose tissues were selected based on *µ_eff_*, *µ_0_* and *BR* results. Specifically, relative differences in *µ_0_* (averaged across kVp), reported as paired values (DM, DBT) were: (−3%, +1%) and (−1%, +4%) for PLA crystal silver and PLA blue versus CIRS with *BR *= 100% (glandular tissue); (+3%, +1%) for ABS white versus CIRS with *BR *= 0% (adipose tissue). According to the *BR* values derived for all acquisition conditions, PLA blue and PLA crystal silver mimicked glandular tissue (*BR*: 101%–109%, 95%–100%, respectively), while ABS white mimicked adipose tissue (*BR*: −2% to 8%). PETG translucent blue and RESIN corresponded to intermediate glandular ratios (*BR* ≈ 50%–70%).

**Conclusion:**

3D‐printed materials sharing the same polymer base can exhibit substantially different attenuation for DM/DBT spectra, resulting in different *BR* equivalences due to formulation variations (e.g., pigments and dopants). Therefore, phantom developers should not rely solely on base polymer designations and declared density when selecting materials for production of breast phantoms; instead, the attenuation of each specific formulation needs to be verified and periodically re‐evaluated under the intended mammography conditions.

## 1 INTRODUCTION

Conventional image quality evaluation in breast X‑ray imaging relies on simple test phantoms with test objects embedded in homogeneous backgrounds, which are well established for routine quality control primarily characterizing the technical performance of the imaging system.^1^ However, this technical image quality assessment does not necessarily reflect the clinical image quality that determines how reliably breast lesions can be detected and characterized.^1^ In addition, digital breast tomosynthesis (DBT) and dedicated breast CT (bCT) were developed to overcome key limitations of digital mammography (DM).^2^ However, the advantages of these modalities cannot be fully evaluated with conventional phantoms, which do not reproduce realistic three‑dimensional breast anatomy or the complex background texture against which lesions must be detected.^3^, ^4^, ^5^, ^6^ Consequently, there is a growing need for anthropomorphic phantoms that reproduce complex breast tissue structures and generate realistic image texture similar to that seen in clinical images.^1^, ^3^, ^4^, ^7^

3D printing technologies are increasingly impacting medical physics through a wide range of applications, from diagnostic radiology and nuclear medicine to radiotherapy.^8^, ^9^ By enabling layer‑by‑layer fabrication of complex geometries from digital models, they offer great flexibility in shape, internal structure, and material selection while remaining relatively inexpensive and accessible. These characteristics make 3D‐printing a cost‐effective production method for anthropomorphic phantoms dedicated to image quality and dosimetry evaluation, among other uses in medical physics.^5^, ^6^, ^7^ Besides achieving realistic anatomical structures, the printing materials must also exhibit X‑ray attenuation properties compatible with the breast tissues, under the beam qualities used in digital mammography (DM) and DBT. Commercial polymers (filaments and resins) employed in fused deposition modelling (FDM) and stereolithography (SLA) are readily available off the shelf, but their detailed composition, additives and pigments are usually not disclosed by manufacturers. These characteristics can influence their X‐ray attenuation, and materials with the same polymer base can present a wide performance range, as investigated in other imaging modalities such as CT.^10^ Several studies have characterized the attenuation of common 3D‑printing polymers such as PLA, PETG and ABS under different X‑ray beam qualities, including CT beams, standardized radiographic spectra (RQR 3–10), mammography spectra and synchrotron‑generated monoenergetic beams.^10^, ^11^, ^12^, ^13^, ^14^, ^15^, ^16^, ^17^, ^18^ In particular, measurements with mammography beam qualities (RQR 2‑M and 4‑M, Mo/Mo 28–35 kVp)^17^ and monoenergetic synchrotron radiation in the 14–36 keV range^18^ have shown that different commercial filaments of the same base polymer can exhibit different attenuation properties.

In a previous study,^19^ an experimental methodology was proposed and validated to determine the effective attenuation coefficients (*µ_eff_*) of a set of 3D‐printed polymers using the clinical X‐ray beams of DM‐DBT systems. In addition, *µ_eff_* values were also determined for CIRS materials (Sun Nuclear, Mirion Medical Co., Melbourne, FL, USA), that are widely accepted as equivalent breast tissue materials.^20^ CIRS plates are widely used as reference materials because they are formulated to reproduce the mass attenuation and glandular ratio (*BR*) of standard breast compositions from *BR* 100/0 (100% glandular) up to *BR* 0/100 (100% adipose) and intermediate *BR* values. Comparing the measured *µ_eff_* values of the 3D printing polymers and CIRS materials provided a rough estimate of the equivalent *BR* for each polymer. For an in‐depth analysis of the influence of beam hardening and scattering properties on the measured *µ_eff_* values, they were fitted to an empirical two‐parameter (*µ_0_, k*) model. Based on the relationships between *µ_0_* and *BR* of CIRS materials, the equivalent *BR* value of the polymers can be derived.^19^

The aim of this work is to investigate the impact of pigments and other dopants used in commercial 3D printing polymers which modify their elemental composition and densities, on the *µ_eff_* values measured for clinical spectra emitted by DM‐DBT systems as well as on their equivalent glandular ratios benchmarked against commercial well characterized breast equivalent materials.

## 2 METHODS

### 2.1 3D‐printing methodology and step‐wedge phantom design

Nineteen commercial polymers from five manufacturers were included in this study representing five polymer classes (PLA‐, ABS‐, PETG‐, HIPS‐based, as well as a photopolymer RESIN) in different color variants (see Table 1). All these materials are commonly used in the production of 3D‐printed breast phantoms.

**TABLE 1:** Set of polymer bases and variants analyzed. Nominal *ρ* corresponds to the density value specified by the manufacturer and measured *ρ* (uncertainty: 0.01 g/cm^3^) to the values measured for printed samples.

Polymer base	Polymer variant (s)	Measured *ρ* (g/cm^3^)	Nominal *ρ* (g/cm^3^)	Manufacturer
PLA	crystal silver	1.23	1.24	BCN3D (BCN3D Technologies Inc. Barcelona, Spain)
	jade white	1.16		BambuLab (Shenzhen Tuozhu Technology Co., Ltd., Shenzhen, China)
	bambu green	1.20		
	pink; black; blue	1.22		
	red; blue grey	1.23		
	grey	1.24		
ABS	black	0.99	1.05	
	white; silver	1.03		
	natural	1.04	1.04	BCN3D (BCN3D Technologies Inc. Barcelona, Spain)
	ABS+ natural	1.05	1.06	eSun (Shenzhen Esun Industrial Co., Ltd., Shenzhen, China)
PETG	translucent blue; red	1.26	1.27	
	translucent grey	1.23		
RESIN	clear	1.21	1.21	Formlabs (Formlabs Inc., Somerville, MA, USA)
HIPS	white	1.03	1.04	Fiberlogy (Fiberlogy SA, Brzezie, Poland)

Step‐wedges of the different polymers were printed using two FDM printers, a BCN3D Sigma R19 (BCN3D Technologies Inc., Barcelona, Spain) and a Bambu Lab X1 Carbon (Shenzhen Tuozhu Technology Co., Ltd., Shenzhen, China). The SLA printer was a Form 3BL (Formlabs Inc., Somerville, MA, USA). For each polymer variant, printing temperature, nozzle diameter, layer height, line width and infill were fine‐tuned to optimize print quality avoiding issues like under‑/over‑extrusion, poor adhesion and nozzle clogging. All FDM polymers were printed with the highest possible infill setting (99% for BambuLab X1 Carbon, 100% for BCN3D Sigma) and using overlapping lines to minimize internal voids. The SLA resin was printed according to manufacturer specifications. Densities of the printed step‐wedges were measured gravimetrically with an uncertainty of 0.01 g/cm^3^ and compared with nominal values provided by the manufacturer (see Table 1).

The step‑wedges were designed with a base area of 1.5×10.0 cm^2^ and eight steps with nominal thicknesses of 0.5, 1.0, 1.5, 2.0, 3.0, 3.5, 4.0 and 5.5 cm, so that multiple thicknesses could be evaluated in a single exposure. The maximum step thickness was selected based on the typically used in the design of breast phantoms (commercial and research‐purpose) for image quality assessment.^1^ Furthermore, this value corresponds to the most frequently reported in both breast screening and dose assessment studies.

### 2.2 DM and DBT imaging

Step‑wedges were imaged individually on a Hologic 3Dimensions system (Hologic Inc., Marlborough, MA, USA), positioning the step‐wedges with the thickest step at the frontal side of the breast support table, where X‑ray fluence is the highest. The images of the step‐wedges were obtained by manually selecting the kilovolts (27, 29, and 31 kVp) in accordance with those chosen by the automatic exposure control (AEC) for compressed breast thicknesses between 3.5 cm and 6.0 cm in clinical practice. This range corresponds to the most frequent breast thicknesses encountered in patient examinations on the system, if the step‐wedge thicknesses are considered. For DM acquisitions, a W/Rh target/filter combination was used with the anti‑scatter grid in place, whereas for DBT a W/Al target/filter was used without the grid. The W/Ag target/filter combination for DM, although available, was not used because a previous work ^19^ showed attenuation values comparable to W/Rh. The appropriate mAs for each material and spectrum was first determined from exposures with the AEC sensor positioned under the thickest step of the wedge. However, for some of the most attenuating polymers and certain acquisition conditions, the mAs could not be selected by the AEC as it exceeded the internally established mAs limits. Thus, for these cases, the position of the AEC sensor was selected under the next‐thickest step. Then, the images of the step‐wedges were acquired manually selecting the mAs value closest to those determined by the AEC. Three for processing 2D images and three DBT series with tube fixed at 0° (quality control mode) were acquired, using only the central DBT projection for analysis. The pixel size for both DM and DBT images was 0.07 mm. For each acquisition condition, additional flat‑field images (no step‐wedge in place) and dark‑field images (2 mm of lead shielding on the detector at minimal exposure) were acquired. All for‑processing images were dark‐field and flat‐field corrected, averaged for each exposure condition and logarithmically transformed.

### 2.3 Effective attenuation coefficients (*µ_eff_*) for 3D‐printed and CIRS materials

Effective attenuation coefficients (*µ_eff_*) were derived from the mean pixel value measured in 0.9 × 1.0 cm^2^ ROIs centered at each step of the logarithmically transformed images. The uncertainty of *µ_eff_* was obtained by error propagation, accounting for the standard deviation of the pixel values within the same ROI and the uncertainty of the measured step thickness. As reference, we use the *µ_eff_* values measured in a previous work ^19^ for CIRS materials with the following glandular/adipose ratios (*BR*): 0/100, 30/70, 50/50, 70/30 and 100/0.

For each 3D‐printed material and exposure condition, *µ_eff_* values were fitted to a two‑parameter empirical model: *µ_eff_ (t)* = *µ_0_ / (1 + k t)* using weighted non‐linear least squares regression analysis. *µ_0_* represents the effective attenuation coefficient for thickness tending to zero and *k* characterizes the decrease rate with thickness of *µ_eff_* due to beam hardening and scatter. Thus, this fit yields an attenuation coefficient *µ_0_* that is independent of material thickness facilitating comparison between different materials, including comparison with the CIRS materials. This model was previously validated for a smaller set of polymers and for the CIRS and PMMA materials using DM/DBT spectra, providing goodness‑of‑fit values *R^2^ > 0.99*,^19^ and was therefore applied in the present work. The robustness of the fits to this model was evaluated by recomputing *µ_eff_* values for each thickness, material and acquisition condition by substituting the corresponding parameters (*µ_0_*, *k*) into this model. The relative differences between the model‑derived and the experimental *µ_eff_* values were calculated across all thicknesses, materials and acquisition conditions.

For the CIRS materials, quadratic relationships of the form *BR = aµ_0_^2^ + bµ_0_ + c* were established for each acquisition condition. The *BR* value of polymers for each target/filter/kVp combination was then obtained by replacing its *µ_0_* value into the corresponding quadratic relationship. The overall uncertainties of the derived *BR* values were obtained by propagating the uncertainties of the fit parameters *a*, *b* and *c*, together with the uncertainty of *µ_0_*.

## 3 RESULTS

### 3.1 Effective attenuation coefficient values of the set of printing polymers and benchmark against commercial materials

**FIGURE 1:**
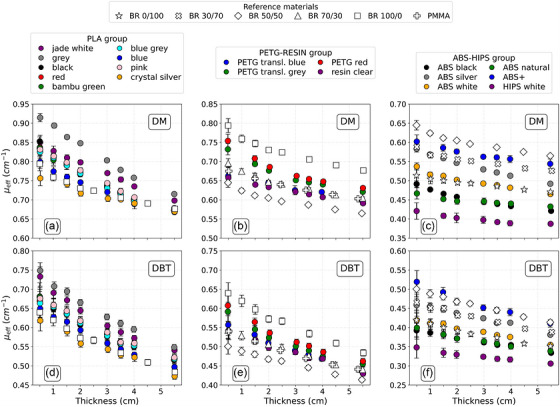
Effective attenuation coefficients (*µ_eff_*) as a function of thickness of reference materials (open markers) and printed polymers (filled markers) at 29 kVp for DM: W/Rh with the anti‐scatter grid in place (a–c) and for DBT: W/Al without the grid (d–f). Error bars correspond to (± *1σ*) bands.

Figure 1 displays the effective attenuation coefficients as a function of thickness for all the 3D‐printed materials evaluated together with the closest equivalent CIRS material, when possible, for benchmarking. As expected, across all DM and DBT conditions, the effective attenuation coefficient (*µ_eff_*) decreased monotonically with thickness for every material, as illustrated in Figure 1 for 29 kVp, with analogous trends observed at 27 and 31 kVp.

The following subsets of polymers are ranked based on their *µ_eff_* values relative to those obtained for the CIRS materials: polymers with *µ_eff_* consistently higher than those of CIRS 70/30 (PLA group), between those of CIRS 50/50 and CIRS 100/0 (PETG–RESIN group), and lower than those of CIRS 50/50 (ABS–HIPS group). Figure 1 also shows that, independently of the thickness, some color variants of 3D‐printed materials with the same base polymer exhibit noticeable differences in *µ_eff_*, particularly for DM spectra. For PLA‐, PETG‐, and ABS‐based materials, the maximum differences between the *µ_eff_* values obtained for DM decreased by 62%, 44%, and 30%, respectively, when the thickness increases from 1.5 to 5.5 cm. In DBT, the maximum differences in *µ_eff_* were lower (27%, 20% and 22% for PLA‐, PETG‐ and ABS‐based materials, respectively).

The highest differences for each polymer group were between for PLA grey and PLA crystal silver, between PETG red and PETG translucent blue, and between ABS+ and ABS black. Furthermore, important differences were identified between *µ_eff_* values of materials sharing the same manufacturer, base polymer and measured printed density (Table 1) with clearly different *µ_eff_* were identified (e.g., PLA black and blue, ABS white and silver, PETG red and translucent blue). In contrast, *µ_eff_* values of some PLA‐based polymers exhibited a discrepancy lower than 5% across all thicknesses and acquisition conditions: PLA bambu green, black, pink, red and blue grey as one subset and PLA blue and crystal silver as another. Plots in Figure 1 show that *µ_eff_* values of PLA crystal silver and PLA blue matched those of *BR* 100/0, ABS white corresponded to those of *BR* 0/100, while PETG translucent blue matched both *BR* 70/30 and PMMA.

### 3.2 Fits to the two parameter (*µ_0_, k*) empirical model

**FIGURE 2:**
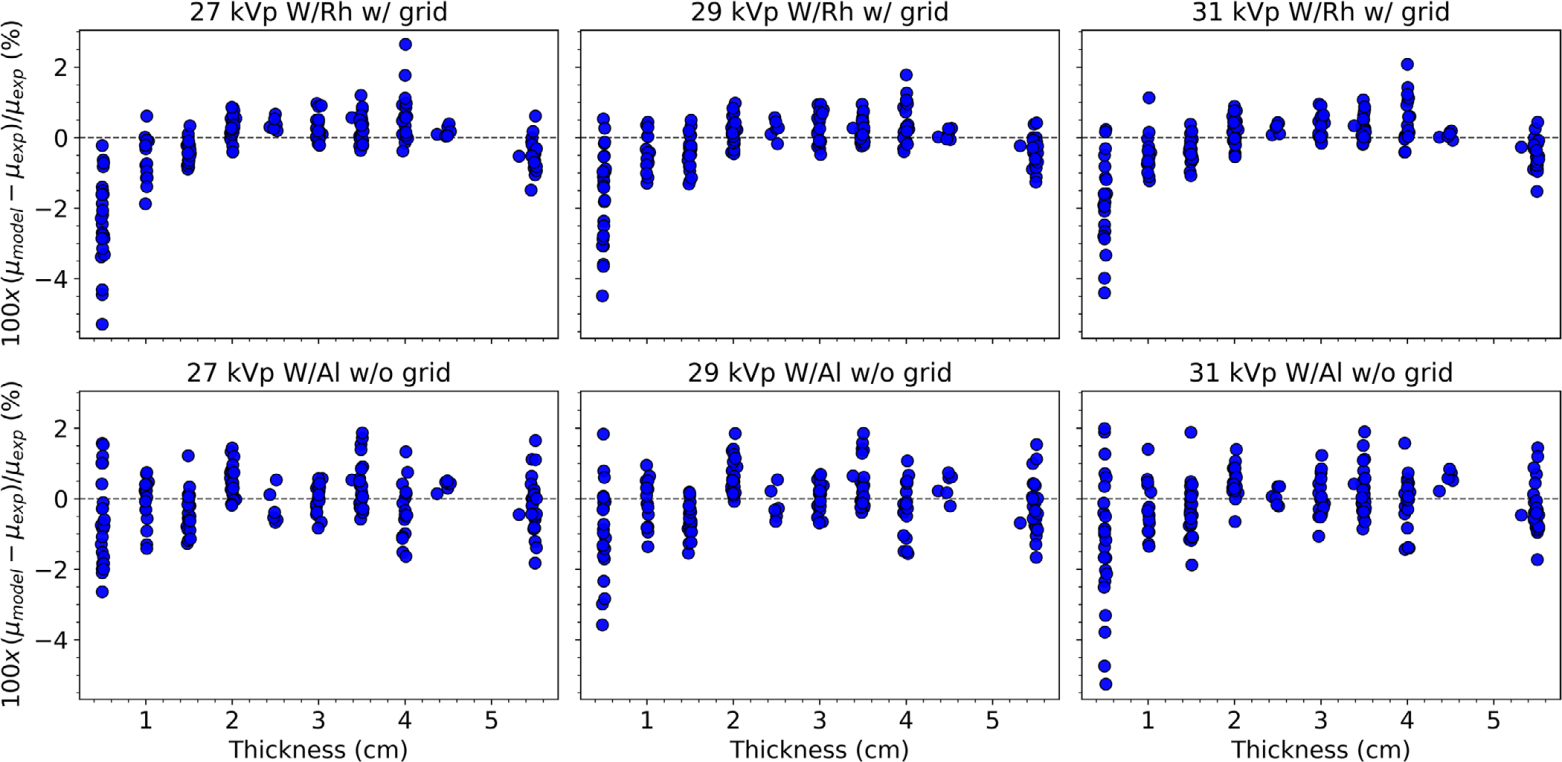
Relative differences (%) between experimental (*µ_exp_*) and model‐derived (*µ_model_*) effective attenuation coefficient values for all thicknesses, materials and acquisition conditions.

Median of *R^2^* values obtained in the fittings of *µ_eff_* to the two‑parameter empirical model are in the range 0.92–0.99 for 112 out of 114 fits. The lowest R2 values were found for HIPS (0.86 and 0.81, for W/Rh @ 27 kVp and 29 kVp, respectively). Over all thicknesses, materials and acquisition conditions, the relative differences between model‑derived and experimental *µ_eff_* values ranged from about −2% to +3% excluding the values for 0.5 cm, as it can be seen in Figure 2. No clear trends were observed with respect to thickness, and no differences were found between DM and DBT or among the different kVp values. For the 0.5 cm thickness, larger discrepancies were observed, with relative differences ranging from −6% to +3%. Overall, for DM acquisitions, the model‐derived *µ_eff_* values tended to be lower than the experimental measurements.

The values for *µ_0_* (*µ_eff_* at thickness tending to zero) derived from the fits decreased by about 17% when switching from DM to DBT for all the polymers. Considering polymers with the same base, maximum relative differences in *µ_0_* were 25%, 10% and 22% for PLA, PETG and ABS considering all acquisition conditions. Values of the parameter *k* (decrease rate of *µ_eff_* with thickness) averaged for all acquisition conditions follow the same order as *µ_0_* (i.e., *k_ABS _< k_PETG _< k_PLA_*). Moreover, when switching from DM to DBT, the corresponding k values were reduced by approximately 30% to 45%, depending on the base polymer.

**FIGURE 3:**
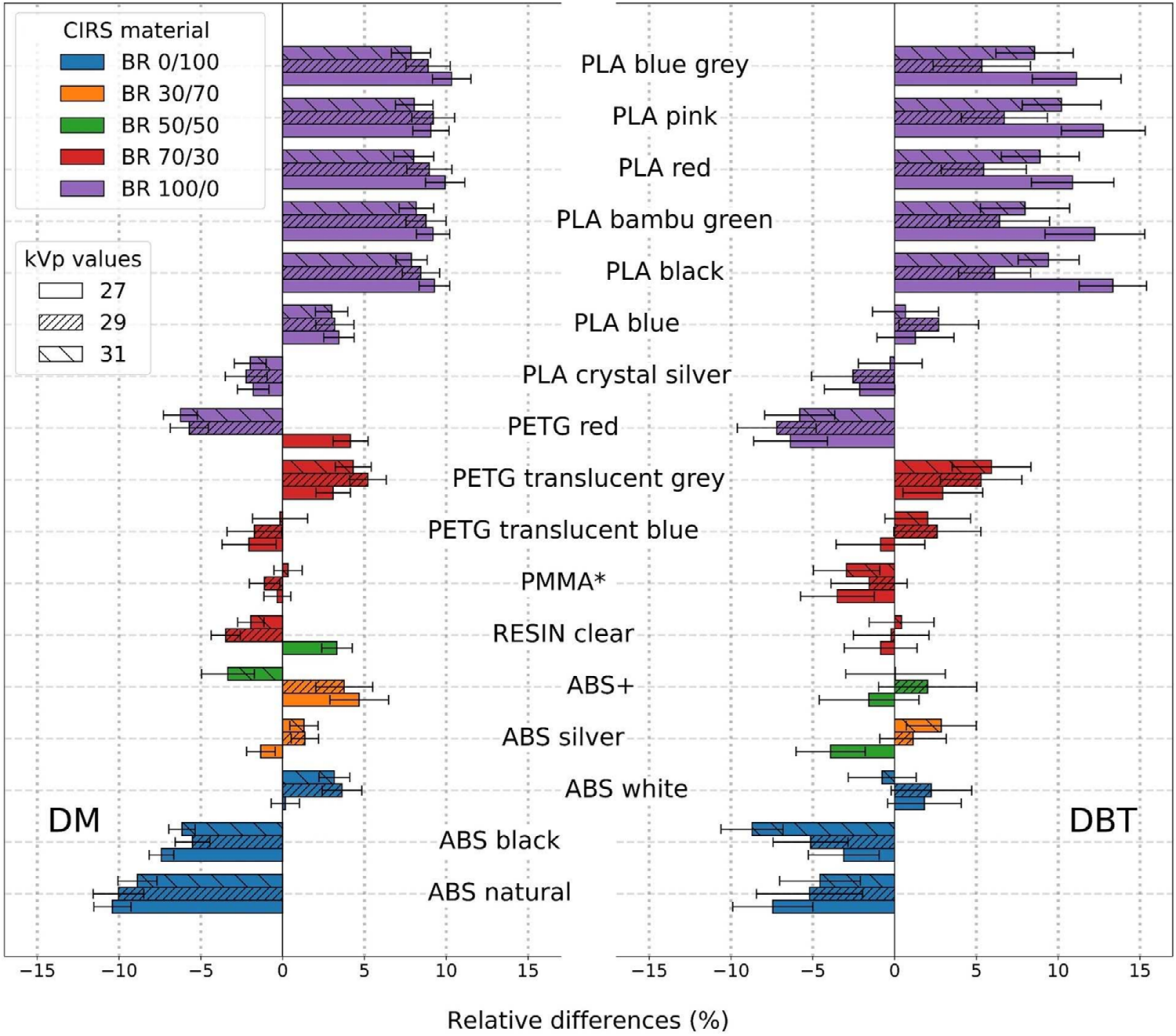
Relative difference between *µ_0_* values of polymers (and PMMA*) and the CIRS materials (bar color) for DM (left) and DBT (right) and for all kVp values (hatch patterns). For each polymer, the smallest relative difference in *µ_0_* with respect to the CIRS reference material is only shown. PLA jade white, PLA grey and HIPS are not included due to their large relative differences. Error bars correspond to (± 1σ) bands.

To determine the equivalence between CIRS materials and polymers, the relative differences in *µ_0_* between each polymer (and PMMA) and each CIRS material were calculated for all acquisition conditions. Figure 3 presents in a bar‐graph, the relative differences of each 3D‐printed material with the nearest CIRS reference material (smallest differences) for each acquisition condition. Such graph facilitates to identify visually the materials that overall are good surrogate candidates for a given benchmarked breast composition for different beam qualities and modality. The bars for 3D‐printed materials exhibiting the largest relative differences (i.e., > +5% from *BR* 100/0 and < −5% from *BR* 0/100) were excluded from Figure 3 to enhance those candidates best mimicking the CIRS materials. This applied to PLA grey, PLA jade white, and HIPS.

As it can be seen in Figure 3, the relative differences in *µ_0_* between most PLA filaments and *BR* 100/0, averaged across kVp, were greater than 8% for DM and ranged from 5% to 13% for DBT. For most ABS filaments, the differences with respect *BR* 0/100 were smallest than 10% and 8% for DM and DBT, respectively. The smallest relative differences (averaged across kVp values) reported in the paired form (DM, DBT) were as follows: PLA crystal silver (−3%, +1%) and PLA blue (−1%, +4%) with respect to *BR* 100/0 and, respectively, making both suitable as glandular‑like surrogates; ABS white (+3%, +1%) with respect to *BR* 0/100, confirming its role as an appropriate surrogate for adipose tissue. In the intermediate attenuation range, PMMA, PETG translucent blue and the RESIN were generally close to *BR* 70/30. The CIRS equivalence of some 3D‐printed materials was spectrum‑dependent: ABS+ matched *BR* 50/50 for W/Rh at 31 kVp whilst was closer to *BR* 30/70 for W/Rh at 27 and 29 kVp (Figure 3); ABS silver corresponded to *BR* 30/70, except for W/Al at 27 kVp, where it matched *BR* 50/50; and the RESIN was consistent with *BR* 70/30 for all conditions except W/Rh at 27 kVp, where it mimicked to *BR* 50/50.

**FIGURE 4:**
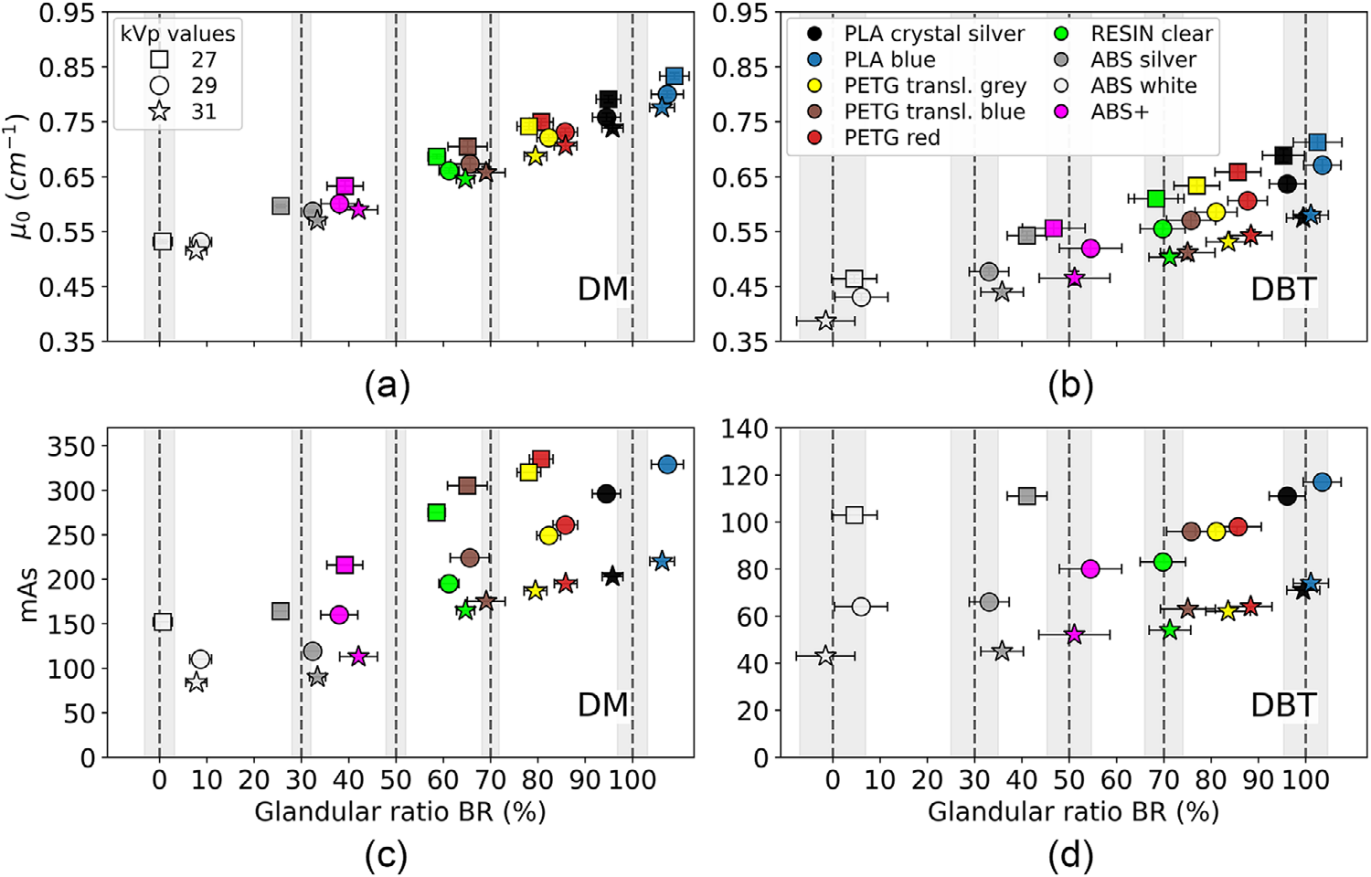
*µ_0_* values (a, b) and mAs selected by AEC (c, d) as a function of the glandular ratio (*BR*) of polymers for DM and DBT. The colour of symbols corresponds to the polymer, and its shape, to the kVp value. Dashed lines indicate the nominal *BR* values of the CIRS materials, and the grey shaded bands represent the uncertainties in their *BR* values estimated from the fits. Error bars correspond to (± 1σ). Most vertical error bars are not visible). The mAs values correspond to those automatically chosen with the AEC sensor under the thickest step.

The equivalent glandular ratio (*BR*) of each polymer for each acquisition condition was deduced from the quadratic fits between *µ_0_* and *BR* (*BR = aµ_0_^2^ + bµ_0_ + c*) of CIRS materials.^19^ The *BR* values of those polymers with *µ_0_* far outside of the *µ_0_* range of CIRS materials were not determined, as the quadratic fits would not be meaningful in these cases. The plots in Figure 4a,b show *µ_0_* values as a function of the derived *BR* of each polymer for DM and DBT acquisitions. The nominal *BR* values of the CIRS materials are plotted as vertical dashed lines. The shaded bands around these lines represent the uncertainty in the *BR* values obtained when *µ_0_* for CIRS are replaced into the quadratic relationship. As can be seen, these uncertainties for DBT were systematically larger than for DM, reflecting the higher *µ_0_* uncertainties, but the overall clustering pattern of polymers relative to the CIRS *BR* ranges is preserved.

The equivalent *BR* values for the selected 3D‐printed polymers are summarized in Table 2, expressed as *BR* intervals covering all tube kVp investigated. Moving from DM to DBT, most polymers showed a shift towards higher *BR* values, indicating a relative increase in glandular‑equivalent attenuation under tomosynthesis conditions, although the magnitude of this shift depended on both the base polymer and even colour. As illustrated in Figure 4, some PLA and ABS colour variants, even from the same manufacturer, product line and in some instances even identical measured density, occupied widely separated positions along the *BR* axis, demonstrating that different polymer formulations can correspond to markedly different breast‑equivalent compositions (e.g., ABS white and silver, PETG translucent blue and red).

**TABLE 2:** Ranges of equivalent glandular ratio *BR* (%) across all investigated kVp values of selected 3D‐printed materials for DM and DBT.

	ABS			RESIN	PET			PLA	
	white	grey	ABS+	clear	blue grey	translucent blue	red	crystal silver	navy blue
DM	1–8	26–33	38–42	59–65	65–69	78–82	81–86	95–96	106–109
DBT	−2–6	33–41	47–57	68–71	68–76	77–84	86–88	95–100	101–103

As expected, the mAs values selected by the AEC to acquire the step‐wedge images increase with the derived *BR* of the 3D‐printed polymers (see Figure 4c,d), reflecting the higher attenuation of the materials according with the AEC functioning of the mammography system of this study.

The results in Table 2 indicate that ABS white behaves as an adipose‑like surrogate, whereas PLA blue and PLA crystal silver were consistent with glandular‐like compositions. Furthermore, ABS grey, ABS+, PETG variants and the clear RESIN could be considered surrogates for intermediate *BR* values.

## 4 DISCUSSION

This study demonstrates that the effective attenuation coefficient (*µ_eff_*) of 3D‐printed materials sharing the polymer base depends on their specific formulation under clinical imaging conditions in mammography and DBT. These values directly measured for different material thicknesses reinforce and extend previous observations of color‑ and additive‑dependent attenuation in nominally identical polymers.^10^, ^11^, ^12^, ^13^, ^14^, ^15^, ^16^, ^17^, ^18^ Polymers are generally grouped into ABS‐HIPS, PETG‐RESIN and PLA according to increasing attenuation, with ABS usually classified as an adipose surrogate and PLA, PETG or photopolymer resins being used as higher‑attenuation materials.^10^, ^11^, ^12^, ^13^, ^14^, ^15^, ^17^, ^18^ However, when working directly with clinical DM/DBT beams and comparing all measurements with well‐established CIRS materials equivalent to breast tissue, it is observed a shift in X‑ray attenuation enough to change the breast‑tissue equivalence assigned to 3D‑printed materials. Therefore, the present work shows that base polymer designation is insufficient for the selection of materials to be used in the production of 3D‐printed breast phantoms.

At small thicknesses, polymers sharing the same base composition showed a large spread in *µ_eff_* values, which indicates that formulation differences (including colorants and dopants) can substantially modulate attenuation under DM conditions. As thickness increases, this spread reduces, particularly for PLA‐based materials, suggesting that the relative influence of pigments and dopants on *µ_eff_* becomes less critical for thicker slabs. Under DBT conditions the thickness‐dependent reduction in *µ_eff_* variability is more moderate and similar across base polymers. This is consistent with the idea that scattered radiation partly damps the impact of colorants on the dispersion of *µ_eff_* values across formulations.

The fit of values to the two‑parameter (*µ_0_, k*) empirical model showed median *R^2^ ≈ 0.94* considering all polymers evaluated and across acquisition conditions. In addition, relative differences between model‐derived and experimental *µ_eff_* values were around 2% for most of the cases (see Figure 3). This assures the robustness of the model as it yields consistent predictions.

On average, switching from DM (W/Rh with grid) to DBT (W/Al without grid) reduced *µ_0_* by about 17%, whereas *k* decreased by approximately 30%–45% depending on the base polymer, which is consistent with differences in effective spectra and scatter conditions between both modalities.^2^, ^10^, ^18^, ^19^ Within each polymer base group, the spread in *µ_0_* (≈25% for PLA, ≈10% for PETG and ≈22% for ABS) is compatible with the effect of pigments, dopants and fillers reported in attenuation characterizations performed with CT and RQR beams.^11^, ^12^, ^13^, ^14^, ^15^, ^17^, ^18^ For example, materials such as PLA black and PLA blue, ABS white and ABS silver, or PETG red and PETG translucent blue displayed clearly different *µ_0_* despite sharing manufacturer, base polymer and measured printed density. This indicates that compositional differences at the formulation level affect X‐ray attenuation.^12^, ^15^, ^18^

The derived *BR* values obtained using the quadratic *µ_0_–BR* relationships for CIRS materials ^19^ provide a direct route to translate polymer attenuation into equivalent breast compositions, clearly seen in Figure 4a–b and Table 2. For the CIRS materials, the differences between the derived‐model and nominal *BR* values range from −2 to 1, considering all materials and acquisition conditions. In addition, the *R^2^* values of the quadratic model were higher than 0.991. The polymer clusters around *BR* values derived from *µ_0_–BR* plots (Figure 4a–b) hold when considering the mAs automatically selected by the AEC of the mammography system. This agreement supports the use of the *BR* values derived from the polymers as a meaningful surrogate for *BR* in terms of AEC response (see Figure 4c,d).

Based on the results obtained for *µ_eff_*, *µ_0_* and *BR* values, despite the number of polymers considered, only PLA crystal silver and PLA navy blue behave as glandular‑like surrogates compatible with *BR* 100/0, whereas PLA grey and PLA jade white exhibit supra‑glandular attenuation. The latter ones are therefore more appropriate as high‑contrast lesion surrogates. ABS white was confirmed as a surrogate for adipose tissue (*BR* close to 0/100), while other ABS variants and HIPS fell below adipose‑equivalent attenuation. They would be more suited to represent structures with lower attenuation than standard adipose tissue. PETG translucent blue showed attenuation comparable to *BR* 70/30 and to PMMA, supporting their use as intermediate tissue surrogates or to introduce background heterogeneities instead of a binary adipose/glandular model.^7^

The *µ_0_* and *BR* values of the 3D‐printed materials facilitates the selection of suitable candidates to produce multi‑material phantoms. The adipose‑, intermediate‑ and glandular‑like components could be chosen from specific, experimentally validated formulations rather than from generic designations such as “PLA” or “ABS”. Furthermore, the experimental methodology based on direct measurements using mammography systems provides a reliable way of assessing the suitability of polymers.

Several limitations should be considered when interpreting the results presented in this study. First, all measurements were performed on a single DM/DBT system using specific W/Rh and W/Al spectra and three kVp settings. Second, only five base polymers and nineteen commercial formulations were investigated, all printed with the highest possible infill setting. Other materials (e.g., nylon, TPU, highly filled composites), alternative infill patterns, or different printing parameters could alter attenuation and, consequently, reported *BR* values.

These limitations nevertheless delineate clear directions for future work that should extend the characterization to additional DM/DBT systems and beam qualities. It would also be valuable to monitor ageing and batch‑to‑batch variability in 3D‐printed materials or phantoms by performing periodic checks of attenuation properties over time and across production batches, to detect potential changes in filament formulation introduced in the manufacturing process.

## 5 CONCLUSION

This work shows that 3D‑printed polymers with the same nominal base composition can exhibit markedly different X‑ray attenuation under clinical DM and DBT spectra, as a consequence of the addition of dopants or colors. Therefore, they must be treated as different materials for breast phantom design. By characterizing nineteen commercial formulations relative to CIRS breast tissue‑equivalent plates, only a limited subset—ABS white (adipose‑like), PLA blue and PLA crystal silver (glandular‑like) and selected PETG/RESIN variants (intermediate)—was found to match specific CIRS glandular ratios, while other color variants within the same product lines deviated substantially from these ratios. These findings imply that phantom developers cannot rely solely on base polymer designations when selecting materials for breast phantoms, but should instead verify attenuation for each specific formulation, and periodically recheck it, under the intended mammography conditions. With the present dataset, multi‑material phantoms can be constructed using validated adipose‑, intermediate‑ and glandular‑like surrogates, and more attenuating formulations can be deliberately assigned as lesion surrogates, thereby supporting more realistic and reproducible task‑based image quality assessment in DM and DBT.

## AUTHOR CONTRIBUTIONS

**Adrián Belarra**: development of the original idea; experimental measurements; analysis of experimental results; writing of the paper; paper revision. **Irene Hernández‐Girón**: writing of the paper; paper revision. **María Castillo**: experimental measurements; writing of the paper; paper revision. **Peter Homolka**: writing of the paper; paper revision. **Margarita Chevalier**: experimental measurements; writing of the paper; paper revision; project management and scientific support.

## FUNDING INFORMATION

This work was supported by the Spanish Ministry of Science and Innovation under grants PID 2021–123390OB‐C21 and PID 2021–123390OB‐C22.

## CONFLICT OF INTEREST STATEMENT

All authors declare no conflicts of interest.

## ETHICS STATEMENT

This work did not involve human participants, identifiable patient data, or animal experiments, and therefore did not require institutional review board or ethics committee approval.
